# 

**Published:** 1898-02

**Authors:** 


### CONTENTS.

ORIGINAL COMMUNICATIONS. PAGE

The Philosophy of Exudative Inflammation. By JOHN ROGERS, M.D., New York . . . 65
Arsenical Necrosis. By OTTO E. INGLIS, D.D.S., Philadelphia . . . . . . . . . 76
X-Rays. By DWIGHT M. CLAPP, D.M.D., Boston, Mass. . . . . . . . . . . . . 86
President's Address. By GEORGE H. CHANCE, D.D.S., M.D. . . . . . . . . . 88
The Funeral of the American Dental Association. By DR. G. ALDEN MILLS, New York . . . . . . . . . . 93

ABSTRACTS AND TRANSLATIONS.

Some Factors which cause Eruption of the Teeth. By DENCER WHITTLES, L.D.S. (Eng.) . . . . . . . . . . 94

REPORTS OF SOCIETY MEETINGS.

American Dental Association . . . . . . . . . . 101
National Dental Association . . . . . . . . . . 112
The New York Institute of Stomatology . . . . . . . . . . 116
Academy of Stomatology . . . . . . . . . . 122

EDITORIAL.

Is Morality a Product of Law? . . . . . . . . . . 126

BIBLIOGRAPHY . . . . . . . . . . 131

OBITUARY.

The American Dental Club of Paris . . . . . . . . . . 134

CURRENT NEWS.

Tenth Anniversary of the Odontographic Society of Chicago . . . . . . . . . . 136
Odontographic Society of Chicago . . . . . . . . . . 136

